# The nature of blindsight: implications for current theories of consciousness

**DOI:** 10.1093/nc/niab043

**Published:** 2022-01-19

**Authors:** Diane Derrien, Clémentine Garric, Claire Sergent, Sylvie Chokron

**Affiliations:** Integrative Neuroscience and Cognition Center, UMR 8002, CNRS & Université de Paris, Paris 75006, France; Institut de Neuropsychologie, Neurovision, NeuroCognition, Fondation Ophtalmologique Rothschild, Paris 75019, France; Inserm, CHU Lille, U1172—LilNCog (JPARC)—Lille Neuroscience & Cognition, University of Lille, Lille 59000, France; Integrative Neuroscience and Cognition Center, UMR 8002, CNRS & Université de Paris, Paris 75006, France; Integrative Neuroscience and Cognition Center, UMR 8002, CNRS & Université de Paris, Paris 75006, France; Institut de Neuropsychologie, Neurovision, NeuroCognition, Fondation Ophtalmologique Rothschild, Paris 75019, France

**Keywords:** blindsight, theories of consciousness, implicit perception, homonymous hemianopia, blindsense

## Abstract

Blindsight regroups the different manifestations of preserved discriminatory visual capacities following the damage to the primary visual cortex. Blindsight types differentially impact objective and subjective perception, patients can report having no visual awareness whilst their behaviour suggests visual processing still occurs at some cortical level. This phenomenon hence presents a unique opportunity to study consciousness and perceptual consciousness, and for this reason, it has had an historical importance for the development of this field of research. From these studies, two main opposing models of the underlying mechanisms have been established: (a) blindsight is perception without consciousness or (b) blindsight is in fact degraded vision, two views that mirror more general theoretical options about whether unconscious cognition truly exists or whether it is only a degraded form of conscious processing. In this article, we want to re-examine this debate in the light of recent advances in the characterization of blindsight and associated phenomena. We first provide an in-depth definition of blindsight and its subtypes, mainly blindsight type I, blindsight type II and the more recently described blindsense. We emphasize the necessity of sensitive and robust methodology to uncover the dissociations between perception and awareness that can be observed in brain-damaged patients with visual field defects at different cognitive levels. We discuss these different profiles of dissociation in the light of both contending models. We propose that the different types of dissociations reveal a pattern of relationship between perception, awareness and metacognition that is actually richer than what is proposed by either of the existing models. Finally, we consider this in the framework of current theories of consciousness and touch on the implications the findings of blindsight have on these.

## Introduction

**Figure 1.**
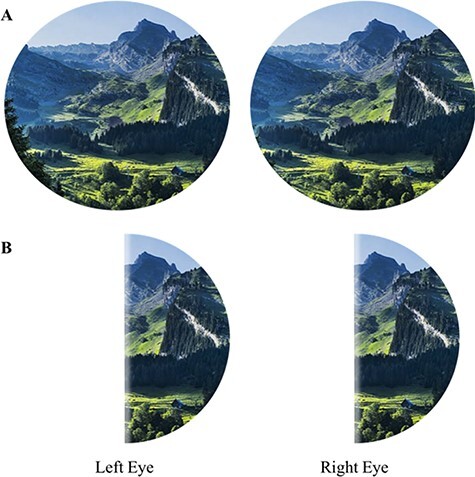
(A) Mountain scenery as seen without visual neurological impairment. (B) Mountain scenery as seen by left and right eye in the case of left HH, caused by right hemisphere brain damage. VFs are approximated

The human visual pathway undertakes processing of the visual sensory modality, transforming light received in the retina into a coherent percept, allowing interpretation of the environment before our eyes. Evidently, a lesion along this pathway has severe consequences. Specifically, post-chiasmatic lesions (i.e. lesions to cerebral structures beyond the optic chiasma, where nervous fibres carrying information from each visual hemifield get segregated) often result in homonymous hemianopia (HH), defined as cortical blindness of the contralesional hemifield usually without ocular damage (Holmes 1918; see Fig. 1 for representation). The most commonly known abnormal perceptual experience in HH patients is blindsight: the manifestation of visual information processing in the blind visual field (VF) of individuals with partial or complete damage of the primary visual cortex (V1) (Weiskrantz *et al.* 1974). The phenomenon was first hinted at by Riddoch (1917) and Poppelreuter (1916), who described, respectively, that hemianopic war veterans sometimes could, in their blind VF, detect moving rather than static stimuli or fill in forms. It was only later, with work on primates, that this phenomenon was further investigated and defined. Monkeys subject to bilateral visual cortex ablation demonstrated some discrimination capacities for orientation, brightness and contrast (Weiskrantz 1963; Pedro *et al.* 1969; Schilder *et al.* 1971; Keating 1975). Quite remarkably, these capacities showed evidence of plasticity as daily training of a monkey lacking V1 allowed it to develop extensive visually guided behaviour (Humphrey 1974). Concomitantly, Pöppel *et al.* (1973) reported patients with V1 damage resulting in contralesional hemifield blindness could perform saccades towards stimuli and thus partially process the blind hemifield. The phenomenon was further brought to light in humans using rigorous forced-choice paradigms (Weiskrantz *et al.* 1974; Sanders *et al.* 1974) in the signal detection theory (SDT) framework for more objective measures of perceptual capacities. The experiments revealed sensitivity for various visual tasks and behaviour despite patient DB reporting no conscious visual experience. The term ‘blindsight’ precisely describes these above chance level discriminatory capacities in forced-choice tasks for stimuli not reported as consciously seen, whilst detection capacities as measured by subjective report remain at chance level (Weiskrantz *et al.* 1974). A few years later, more extensive studies on DB and studies of another patient with blindsight (GY) additionally identified occurrences in which patients reported some awareness of the visual stimuli, but that this experience was different from actual visual perception (Weiksrantz *et al.* 1995). This revealed that blindsight is more complex than first thought: it is composed of multiple aspects that are differentially impacted in patients.

To understand how such a phenomenon arises, researchers interested in blindsight have made use of technological progresses to delve into its neuroanatomical correlates. Indeed, theories of neurological (dys)functioning, however well developed, cannot overlook the neuroanatomy and associated brain activity of neuropathological subjects. Moreover, these studies have provided extremely valuable insight into the implementation of visual perception and how, from its damage, blindsight can arise. In addition, the study of blindsight cannot be dissociated from the study of consciousness: a valid theory of consciousness must be able to predict the occurrence of blindsight phenomena following the structural damage these patients have endured. The ‘abnormal’ phenomenological experience of these patients must be coherent with how consciousness in neurotypical individuals arises.

In this article, we aim to investigate blindsight through the lens of theories of consciousness as well as acquire, from the behaviour of these patients, a better understanding of the mechanisms of consciousness in general.

### Structural and functional neuroanatomy of blindsight

**Figure 2.**
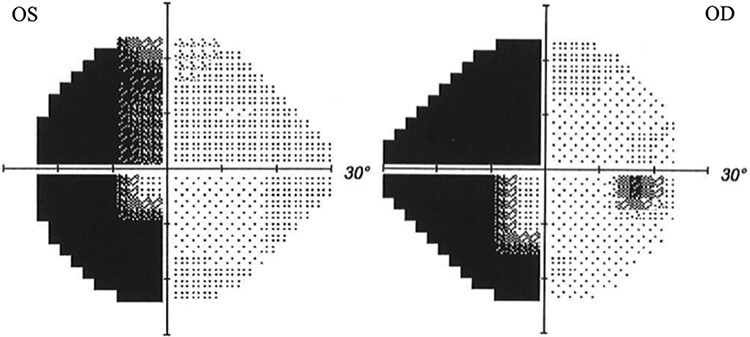
Example Humphrey Field Perimetry of a patient with left HH with macular sparing. OS—Left eye. OD—Right eye

Post-chiasmatic damage in the visual pathway significantly alters visual consciousness: clinical blindness of the contralesional hemifield is the functional defining symptom of hemianopia revealed by ‘tasks of brief stimuli’ detection such as the VF perimetry (Fig. 2). Normal vision relies for the most part on visual information reaching V1 before further processing in the extrastriate cortex. However, the visual system is made up of far more pathways. For instance, evidence from primate studies suggest visual information reaches the frontal eye fields in the frontal cortex (Schmolesky *et al.* 1998). Some visual information is transmitted by specific retinal ganglion cells projecting (1) to the ventral lateral geniculate nucleus (LGN), which relays information to extrastriate areas (the geniculo-extrastriate pathway), notably the middle temporal area (MT) and (2) to the superficial superior colliculus (SC), onto the pulvinar with direct connections to extrastriate areas (the retinotectal pathway; Adams *et al.* 2000; Baldwin and Bourne 2020). Importantly, these subcortical pathways undertake visual processing that is not necessarily available to awareness (e.g. visual saccades; Spering and Carrasco 2015). It is also worth noting these routes have an important role during the development of the visual system (Mundinano *et al.* 2018). Identifying the neuroanatomical damage underlying the loss of vision in half the VF, as well as the pathways potentially associated with the remaining function provides valuable information for understanding the implementation of said function. These visual routes that bypass V1 are strong candidates for subserving blindsight (Rima and Schmid 2020).

#### Alternative substitute pathways

Sanders *et al.* (1974) first suggested blindsight is predominantly mediated by existing visual pathways from the retina to posterior association areas via the SC. The role of the SC in the mediation of blindsight has been supported by studies in macaques (Mohler and Hurtz 1977; Rodman *et al.* 1990; Kato *et al.* 2011) and humans (Tamietto *et al.* 2010; Leh *et al.* 2010; Georgy *et al.* 2016) since. Danckert and Rossetti (2005) also propose the processing of stimuli in the blind VF relies on either the retinotectal pathway or the geniculo-extrastriate pathway, which both bypass V1. They defend the behavioural manifestations depends on which terminations of these pathways remain (partially) functional in each individual. Patients able to perform actions (pointing, saccades etc.) relative to stimuli in their blind VF rely on the ‘automatic pilot’ subserved in part by retinotectal terminations to dorsal extrastriate cortex and posterior parietal cortex (Rossetti and Pisella 2002; Danckert *et al.* 2003). Functional imaging of patient GY performing such actions indeed showed activity in dorsal and not ventral visual regions during these tasks (Baseler *et al.* 1999). In addition, Kato *et al.* (2011) showed the retinotectal pathway is crucial for visually guided saccades after V1 damage. Macaque studies highlight the involvement of the pulvinar as a relay (Clower *et al.* 2001; Berman and Wurtz 2010; Lyon *et al.* 2010). Moreover, both its inhibition and blocking its input from the SC results in decreased blindsight-associated performances in two macaques (Kinoshita *et al.* 2019). In patients capable of attentive processing in the blind VF (‘attention-blindsight’, e.g. Kentridge *et al.* 1999), the underlying functional region is suggested to be MT, itself receiving SC input and involved in high-level visual processing. Finally, patients whose residual processing can only be made evident in forced-choice conditions, without awareness (‘agnosopsia’, e.g. Zeki and Ffytche 1998), manifest residual function of the geniculo-extrastriate pathways from dorsal LGN to ventral extrastriate areas (Sincich *et al.* 2004), involved in the ‘what’ of vision. The LGN’s key role is emphasized by its cells maintaining transmission of visual information after V1 lesion in marmosets (Yu *et al.* 2018). Also, its reversible inactivation in monkeys with V1 ablation prevented the brain activations and behavioural responses following stimulation of the blind VF (Schmid *et al.* 2010). Evidence in humans comes from functional imaging studies in hemianopic patients, which indicate the involvement of the LGN to extrastriate projections during occurrences of blindsight behaviours (Ajina *et al.* 2015; Ajina and Bridge 2018).

Interestingly, these pathways appear to be nonconscious components of vision, parallel to pathways for visual perception in healthy individuals (Spering and Carrasco 2015). For instance, SC connections to MT are believed to subserve pursuit eye movement, which is a directed movement, dissociated from awareness (Spering *et al.* 2011). It thus appears that blindsight behaviours rely on existing pathways in the visual system, and that these pathways are by default dissociated from awareness, which nonetheless have strong interconnections with visual pathways involved in awareness (Goodale and Westwood 2004).

#### Functional and anatomical remodelling

Tractography and functional imaging studies highlight the role of neuroanatomical plasticity, suggesting novel or strengthened connections might potentiate behavioural capacities. Functional studies of GY showed activity in ipsilesional MT during motion detection in his blind hemifield, without V1 activation (Barbur *et al.* 1993). Note here that GY suffers from HH due to an occipital lesion resulting from a traffic accident at a young age, perhaps facilitating plasticity mechanisms compared to post-stroke HH patients who tend to be older. Nonetheless, Bridge *et al.* (2008) showed strengthened connections to ipsilesional MT as well as novel bilateral MT connections and later showed normal activity in ipsilesional MT after stimulus presentation in the sighted VF (Ajina and Bridge 2017). Tractography analysis on 17 patients with V1 damage showed that those with blindsight always had intact LGN to MT connections (Ajina *et al.* 2015). Diffusion-weighted imaging showed that two out of four attentional blindsight patients displayed reorganized outputs of ipsilesional SC with strengthened and novel connections to contralesional visual areas (Leh *et al.* 2006). The researchers argue that these results go in favour of the unconscious vision theory (discussed further down) as these nuclei (SC, LGN and MT) belong to pathways ‘not sufficient’ for conscious visual information processing (Blake *et al.* 2014).

Evidently, there are some architectural differences between the implementation of normal vision and of residual capacities in hemianopics, thus making them different from normal, degraded vision. Simultaneously, it is unmistakeable that the functional deficit is not akin to total abolishment of visual information processing in the hemifield declared blind according to the visual perimetry. We next describe the different phenomena of blindsight, which have been manifested in HH patients.

### Unconscious capacities and phenomenological experience

The two main components to blindsight behaviour are objective performance on a forced-choice task and subjective awareness rating. Objective capacity refers to the capacity to make factual and accurate perceptual decisions, irrespectively of perceptual experience; subjective awareness is derived from subjective reports that allow us to determine if a percept was conscious or not. The subtype of blindsight is defined according to the performance in ‘both’ of these aspects. Blindsight type I is defined as being able to successfully, yet without perceptual awareness, discriminate targets in the blind hemifield; it is the complete dissociation between conscious awareness and objective capacities. In blindsight type II, not only are these objective discriminatory capacities present, they are also accompanied by some phenomenological subjective experience: patients have ‘feeling’ in their blind VF and thus report some form of awareness (Weiskrantz 1998). Finally, blindsense is defined as having phenomenological experience specifically linked with the presence of a stimulation in the blind field, but in the form of a ‘non-visual’ experience, and showing no objective capacities in forced-choice tasks, neither for binary visual detection nor for discrimination (Garric *et al.* 2019). The critical aspect of this phenomenon thus relates to the terms used for reporting one’s introspection: these patients will acknowledge ‘feeling the presence of a stimulus’ but will deny it the status of ‘visual’ experience. This nuance has been overlooked in previous methodologies used for prompting introspection in these patients. By including the term ‘sensation’ and distinguishing it from ‘vision’ in our subjective awareness scale (SAS), we are able to capture this phenomenon: blindsense patients can accurately report feeling the presence of stimuli presented to their blind VF yet have no objective visual sensitivity as assessed by forced-choice tasks. Note that Garric and colleagues designed the SAS guided by how HH patients ‘themselves’ describe their impairment in clinical or rehabilitative contexts. Indeed, the notion of ‘feelings’ or ‘sensing’ in the blind VF, again ‘different’ from normal vision and perhaps even different from an experience we could consider as visual, is overwhelmingly present in the reports from these patients. The hypothesis is that the functional consequence of damage in the visual cortex could in a sense disrupt the visual component of the subjective sensation originating in this area. The SAS allows to assess the qualitative experience of the patients’ blind VF. Subjects select the level that describes best their subjective awareness during a trial, notably with an increment not referring to the visual modality ((i) ‘I did not see anything’; (ii) ‘I don’t think that I saw anything, but I am not sure’; (iii) ‘I felt something’; (iv) ‘I saw something’; and (v) ‘I clearly saw something and can identify it’). Subjective sensitivity was calculated using the area under the receiver operator characteristic curve, which provides a statistical measure of the difference in rating for signal and noise trials. They showed that a majority of patients accurately reported sensation along the SAS (at differing levels) relative to stimuli occurrence. ‘Blindsense’ is thus a degree of correct (phenomenological) nonvisual subjective performance in response to a stimulation of the blind VF, whilst classical visual objective discrimination remains at chance level—i.e. the patient has no real conscious visual access allowing for the processing required by discrimination tasks. This differs from blindsight in which subjective performance is at chance and objective performance is significantly above theoretical chance level. Blindsense is the phenomenon of ‘sensing’ stimuli in the blind VF without seeing them, a phenomenon frequently described by patients in clinic. This novel dissociation was seen in 50% of their cohort and was perhaps missed in previous studies, as patients can be reluctant to assert that they ‘feel’ rather than ‘see’ something in their blind VF if not given the option. It is important to note blindsense remains a debated concept and needs further experimental confirmation (see Phillips 2021a; Garric *et al.* 2020).

Blindsight studies have revealed the subtle evidence that residual activity along the visual pathway may translate into objective capacity and/or subjective sensitivity during forced-choice visual tasks in the blind VF. In parallel, studies that have focused on the methodology employed to expose these capacities highlight the importance of task design (Mazzi *et al.* 2016; Garric *et al.* 2019). From these, two main theoretical conceptions of blindsight phenomena arise.

### Models for underlying mechanisms of blindsight

The subtle yet crucial differences between these dissociation phenomena have led to two differing hypotheses of their underlying nature. Some argue blindsight is in fact ‘degraded vision’, whereas the contrasting opinion is that blindsight reveals ‘unconscious’ capacities.

#### Blindsight is qualitatively degraded vision

Soon after the first studies on blindsight, Campion *et al.* (1983) argued that they could not be considered as evidence for unconscious vision. These authors rather explain the lack of conscious experience in these patients through a shift in their criterion for reporting awareness. The conscious experience of the patients is severely degraded, making them unlikely to naturally report it. This theory claims that hemianopics tend to have extremely conservative response criteria for visual tasks and defend the behaviour defined as blindsight stems from the instability of such criteria (see Phillips 2021b for more information on this theory). Contenders of this model argue that reports of objective visual performance in the blind VF come down to a conservative shift in response criterion and ‘degraded phenomenal awareness is present [and] is unacknowledged’ (Phillips 2021b). Some patients achieve better performance in tasks that ask to report when ‘aware’ of a signal than in those instructing to report ‘seeing’ a signal (Stoerig and Barth 2001; Weiskrantz 1980; patient EY). This model defends that these findings show how instruction design modulates the response criterion; they do not indicate subjective awareness despite lack of vision. Mazzi *et al.* (2016) describe the case of a patient who is only accurate on trials in which she reports some awareness on the perception awareness scale (PAS; Ramsøy and Overgaard 2004), drawing the conclusion that accurate judgement in the blind VF relies on a kind of conscious vision. They argue this patient shows that indication of a weak experience on the PAS corresponds to her reporting a ‘guess’ on a binary scale and thus questioning awareness rather than sight invites the participant to lower their response criterion, thereby allowing to expose their perceptual experience (Mazzi *et al.* 2019). The degraded vision theory therefore implies performance considered objective increases when awareness is reported (Phillips 2021b).

#### Blindsight as a manifestation of unconscious vision

The alternative model argues blindsight is the manifestation of ‘unconscious processing’, as objective responses to stimuli in the blind VF resemble automatic behaviour (Danckert and Rossetti 2005). Even when performance levels are matched between the sighted and blind hemifields, patients with diagnosed blindsight will report awareness for stimuli in their sighted VF but not for those in their blind VF (Persaud *et al.* 2011). Moreover, blindsight patients achieve better sensitivity in two-alternative forced choice (2AFC) discrimination tasks than in yes–no (YN) detection tasks (even following appropriate mathematical corrections; Azzopardi and Cowey 1997), indicating some information is exclusively available for discrimination, not detection (Azzopardi and Cowey 1997). This discredits the account by which a change in response bias between the two tasks is responsible for the change in performance and implies the information exclusively available for discrimination is not conscious (Weiskrantz 2008). In the same vein, Persaud and Cowey (2008) explored higher-level cognitive processing in patient GY by administering an exclusion task (inspired from process dissociation procedures; Jacoby 1991) in which the participant has to respond the opposite of what the stimulus shows (e.g. if an arrow points left, the participant should respond ‘right’). Interestingly, although patient GY successfully (yet without awareness) performs a classic task, he performs at chance level in the exclusion task, seemingly indicating the visual information is not processed consciously as it is not available for higher-order cognitive levels (Persaud and Cowey 2008). It is worth noting this conceptualization of blindsight also points out a severe, yet incomplete, impairment of visual sensibility and explains the blindsight behaviour partly because these patients adopt an extreme response criterion (Azzopardi and Cowey 2001). As a matter of fact, in trying to understand ‘why blindsight is blind’, these authors propose that damaging V1 induces a contralesional VF in which visual sensibility is severely impaired, although not completely abolished. We agree with Azzopardi and Cowey’s proposition that patients with blindsight are blind to stimuli in their hemianopic field partly because they adopt an extreme response criterion. Indeed, in the frame of SDT, this could be interpreted in terms of loss of sensitivity. However, as Azzopardi and Cowey (2001) discussed at the end of their paper, and in agreement with Weiskrantz’s (1986) view, psychophysical experiments, at least with static targets, show that response bias may not account for ‘all’ of the difference between YN and 2AFC performance. Along those lines, visual processing in the impaired field should not be seen as unusual. As Azzopardi and Cowey (2001) conclude, the discrepancy between YN and 2AFC tasks could be caused by an impairment of decision-making process due to the disruption of neural mechanisms responsible for optimizing the response criterion during psychophysical protocols. Indeed, the task instructions are likely to influence the response criteria in such a way that it dissociates from the patient’s perceptual criterion, causing it to jitter. This view is compatible with the unconscious vision theory as correct responses, thanks to response criterion jitter does not necessarily indicate that patient consciously perceived the stimulus, as the perceptual criterion is likely to remain extremely conservative (Michel and Lau 2021). Advocates of this conception of blindsight argue that it has a great impact on the theories of visual consciousness (LeDoux *et al.* 2020).

As seen above, the major contending theories of manifestations of vision in HH patients are that (i) these phenomena are either abnormal or degraded conscious vision and they occur because the protocols manage to lower the highly conservative response bias in these patients or (ii) these phenomena are indications of unconscious vision. We believe looking at these behaviours through the lens of theories of consciousness will bring rich insight into how they can arise following structural damage to V1.

## Blindsight through the prism of two modern theories of neurotypical consciousness: global neuronal workspace and recurrent processing theory

Contenders of the degraded vision interpretation of blindsight argue that these patients are experiencing degraded yet normal vision rather than showing capacities of perception without awareness. Conversely, proponents of the unconscious vision interpretation believe the pathways remaining (partially) functional enable some information processing but that the damage prevents the synchronized activity facilitating conscious vision (Melloni *et al.* 2007). In what follows, we examine how these two interpretations might be implemented within two main contemporary models of consciousness: the global neuronal workspace (GWS) model and the recurrent processing theory (RPT). Indeed, these two models appear to bring complementary perspectives that can help understand different facets of the blindsight phenomenon.

### Blindsight through the GWS prism

Conscious processing or a conscious representation is often defined as being reportable by the subject (Sergent and Naccache 2012), as opposed to the neural processing that cannot be accessed and is thus referred to as ‘non-conscious’. For the past decades, the origin of consciousness, its dynamics, its neural correlates and much more have been thoroughly studied in order to disentangle sensory processing from conscious access processing (Aru *et al.* 2012; Koch *et al.* 2016). One major contender is the GWS theory that states that sensory input will become a conscious representation so long as it generates a global, synchronized brain activity, sufficiently sustained in time (Baars 1989, 2005; Dehaene *et al.* 1998). This implies that the activity resulting from sensory input, initially locally contained in its corresponding sensory cortex, must be sufficient to ‘ignite’ the brain globally, in particular the prefrontal cortex (Dehaene and Naccache 2001). Focusing on the visual modality, the GWS asserts it is the synchronization between visual and higher-level multimodal areas through the fronto-cingulo-parietal network that allows visual signal amplification necessary for conscious perception (Dehaene and Naccache 2001; Sergent and Dehaene 2004a). It is important to note that other theories of consciousness defend that activation of prefrontal areas in response to visual input is essential for visual consciousness, such as the higher-order thought model of consciousness (Rosenthal 2019). To understand the dysfunction at hand regarding the visual sensory modality in hemianopics, one should refer to the conception of visual consciousness in the neurotypical individual. Damage to V1, classically thought to be essential for visual consciousness (Stoerig 2006), is believed responsible for this lack of synchrony, which impairs the connection from visual to prefrontal areas. We now explore how the previously presented accounts of blindsight behaviour may be implemented in the GWS theory of normal consciousness.

According to the degraded abnormal conscious vision theory, behaviours revealing processing of the blind hemifield are the result of activity along the normal vision pathways, which are damaged yet functional to a certain extent. If reasoning in terms of the GWS, this weak residual connectivity in the visual system would be insufficient to generate either global ignition of the brain or sustained synchronous activity, which are both essential for conscious access. Thus, visual stimulation in the blind VF is incapable of producing appropriate activation of prefrontal areas. The GWS also postulates activation of prefrontal areas following sensory input is implicated in decision-making (Dehaene and Changeux 2000). The weak residual connectivity therefore is insufficient for visual input in the blind VF to generate enough activity to overtake the decision criterion for reporting a visual experience. Indeed, contenders of the abnormal vision theory defend that patients may not report visual consciousness or even awareness due to conservative decision criteria (Phillips 2021b).

It should be noted that the fact that observers report they do not see a stimulus may not mean they have absolutely no subjective experience; they may be giving such reports in relative terms in the context of other stimuli. This could indeed be the case of patients with blindsense (Peters *et al.* 2016). Nonetheless, that is not to say this subjective experience is sufficient to generate a normal visual experience or full-blown conscious access (Michel and Lau 2021). In addition, Peters *et al.* (2016) pointed out the fact that because blindsight patients are rare, there have been attempts to recreate blindsight in healthy subjects. However, these studies have also highlighted the ongoing controversy regarding the relationship between metacognitive sensitivity (i.e. correspondence between confidence and accuracy) and conscious awareness in relative blindsight (reconstruction of blindsight in healthy subjects). In addition, relative blindsight has been found to be susceptible to the contamination of response bias. Indeed, relative blindsight refers to the phenomenon that, for similar stimuli at identical objective task performance levels (e.g. accuracy in stimulus discrimination), healthy observers can have different subjective levels (or frequency) of reported awareness in different conditions. As Peters *et al.* (2016) argue, if we are concerned with the subjective rather than the objective aspects of perception, criterion bias may well be the very measure we should focus on and we should not try to avoid this question. In this way, the GWS should include the question of a distinction between perceptual and decision criteria as proposed by Peters *et al.* (2016). Bias-free signal detection theoretic measures provide an excellent method for avoiding response bias confounds, and many researchers correctly adopt this approach. However, here we discuss how a fixation on avoiding criterion effects can also be misleading and detrimental to fruitful inquiry. In a recent paper, Balsdon and Azzopardi (2015) claimed that contamination by response bias led to flawed findings in a previous report of ‘relative blindsight’. We argue that their criticisms are unfounded. They mistakenly assumed that others were trying (and failing) to apply their preferred methods to remove bias, when there was no such intention. They also dismissed meaningful findings because of their dependence on criterion, but such dismissal is problematic: many real effects necessarily depend on criterion. Unfortunately, these issues are technically tedious, and we discuss how they may have confused others to misapply psychophysical metrics and to draw questionable conclusions about the nature of transcranial-magnetic-stimulation (TMS)-induced blindsight. We conclude by discussing the conceptual importance of criterion effects in studies of conscious awareness: we need to treat them carefully but not avoid them without thinking.

**Figure 3.**
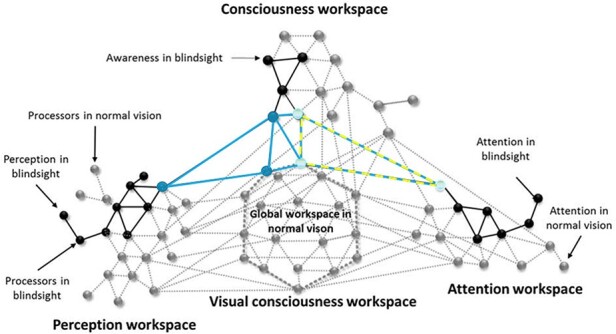
Blindsight in the GWS. Black circles/lines represent remaining functional processors mediating blindsight, which do not include enough long-range connections for synchronized activity. Blue circles/lines represent processors thought to mediate awareness in blindsight type II. Subjective awareness in blindsense could be linked long-range connections between consciousness and attention workspaces, represented by light blue circles and yellow lines. Adapted from Hadid and Lepore (2017)

The implementation of blindsight in the GWS taking the stance of the unconscious vision model was described by Hadid and Lepore (2017), who argue that the phenomena of blindsight rely on ‘some’ functioning components of the pathways for visual consciousness. They are mediated by local loops remaining functional, but the HH-causing damage impacts the connections allowing these local loops to ignite the global workspace necessary for visual conscious access (Fig. 3). Similarly as above, highly conservative decision criteria may arise in patients as a consequence of this dysregulated activation following visual input (Cowey 2010). Conversely, reports of some degree of awareness are argued to be evidence of partial functioning of the loops of the awareness workspace (Hadid and Lepore 2017). Functional magnetic resonance imaging evidence of dorsolateral frontal cortex activity exclusively in blindsight type II, and not type I, suggests the fronto-parietal network is key for awareness (Sahraie *et al.* 1997), as predicted by the GWS. Ko and Lau (2012) further build on this theory and propose that the conservative criteria, poor detection capacities and low and inaccurate confidence ratings of blindsight patients stem from a disruption in their metacognitive system, which relies on the prefrontal cortex (Lau and Rosenthal 2011).

We have shown both models of blindsight mechanisms can be implemented in the GWS framework, which is arguably one of the major explanations of the neural origin of consciousness. Next, we consider the role of local visual feedback connections in blindsight, in line with the RPT, which defends that they are both necessary and sufficient for conscious visual perception (Lamme 2010). One of the main differences between RPT and GWS theory is that the GWS theory considers that even when recurrent loops occur between visual areas, this processing can still be unconscious if it is not connected to a wider network of areas, critically including the attentional network.

### Recurrent connections and blindsight

The RPT crucially relies on the distinction between two stages that can be distinguished during sensory processing of an external stimulus: (i) visual stimulation creates a feedforward sweep of information, during which visual areas process each specific object features (e.g. borders) in an unconscious manner and (ii) once higher-order visual areas achieve the processing and integration of the more complex visual features, they feedback this information to lower-level visual areas, such as V1, through recurrent connections, and this generates conscious visual perception. Lamme explains the objective residual capacities in blindsight by suggesting they are the result of the first stage, the feedforward sweep of information, but that due to the V1 lesion no phenomenological consciousness may arise (Lamme 2001). This inherently relies on pathways that bypass V1, such as the previously mentioned retinotectal and geniculo-extrastriate pathways. Indeed, these routes enable some processing of relevant visual features as 20–30% of extrastriate cells remain responsive following V1 ablation in macaques (Schmid *et al.* 2009, 2010, 2013). Moreover, cells already show evidence of tuning during the feedforward sweep at ∼150 ms (Thorpe *et al.* 1996). The RPT defends that visual perception necessitates the integration of a stimulus within its context, a process achieved by recurrent connections within the visual cortex, which enable specific receptive field responses being tuned ‘within their context’. Blindsight is thus the manifestation of the disruption of the recurrent connection process, as V1, an important hub of integration of these loops, is damaged. In the framework of conscious access being necessary for perceptual experience, the RPT framework strongly argues against the degraded vision theory of blindsight. Rather, it defends that some HH patients can process specific features of visual stimuli thanks to routes bypassing V1 to reach extrastriate areas but are not aware of the processing as it cannot be integrated within the entire VF. This nicely explains feature-specific objective capacities of blindsight, which tend to be low-level features (note that residual capacities concerning the processing of emotions—a higher-order feature—are likely to involve visual information directly reaching the amygdala and temporal cortex; Lamme 2001). However, this does not explain how residual subjective perception can occur in HH patients (i.e. blindsight type II or blindsense). Moreover, although beyond the scope of this article, we would like to point out that in the framework of perceptual consciousness overflow (Lamme 2010; Block 2011), residual recurrent loops in hemianopic patients could be capable of mediating consciousness without conscious report and thus be reconciled with the qualitatively degraded conscious account of blindsight.

Recurrent theories of awareness could be reconciled with this observation if we consider that, although V1 is the main player in generating normal visual awareness in this theory, it is likely to not be the only player. Indeed, V2 has been suggested to also be involved in visual awareness (Salminen-Vaparanta *et al.* 2012). Notably, the first model of RPT posited that V2 may be involved with medium-grain visual awareness (Pollen 1999). Hence, perhaps V2 can have the potential to subserve some sort of visual perception, if the lesion permits information to reach V2. Importantly, the subjective perception described by some hemianopic patients tends to be rather non-specific, often referred to as the ‘feeling that something is there’ as opposed to normal visual sensation. GY states he does not ‘ever sense anything […] it is more an awareness but you don’t *see* it’ (Weiskrantz 1997). However, it should be reminded that V2 is not necessary in visual awareness, since its ablation does not result in major visual awareness dysfunction (Merigan *et al.* 1993). Moreover, this explanation implies there are surviving recurrent loops to V2, allowing V2 to generate ‘medium-grained’ awareness for subjective experience. There is to this date no evidence of such connexions, hence this is not a satisfactory explanation of blindsense.

### How far is blindsight really from neurotypical consciousness?

There is to this day no consensus on the underlying mechanisms of ‘blindsight’. However, it is undeniable that a certain proportion of patients diagnosed with a VF defect have behaviours that are evidence that there can be, to varying extents, some processing of visual information in the blind hemifield. The structural damage nonetheless does result in functional impairment and neurological rearrangement, making any remaining function in essence different from normal vision. Crucially, this conception stems directly from the reports of blindsight patients, who describe their contralesional hemifield as being inexistent, often referring to it as akin to the back of their head. In practice, the damage ‘reassigns’ the borders of the normal VF to the ipsilesional hemifield, thus making the patients effectively clinically blind in the other hemifield. However, blindsight behaviour is evidently not constant across all conditions, suggesting many variables can manipulate it. For instance, the report of visual sensation and the ability to visually discriminate are influenced by instruction design, stimulus characteristics and repetitions that modulate the response criterion and can perhaps also act on sensitivity. We believe that these influences are evidence that hemianopia may result in a perturbed visual system that retains some preservation of information processing. Things such as the design of tasks or prior beliefs are likely to impact the baseline activity of the some (specific) regions involved in the process of visual perception tasks. We believe these modulations highlight the wide spectrum of altered visual experience observed in hemianopic patients, which includes blindsight and its subtypes.

## Altered residual functioning: a multidimensional spectrum of phenomenology in hemianopics

To summarize our previous analysis, we suggest HH-causing damage induces an alteration of the connexions between the visual system and the GWS, resulting in atypical phenomenological experience and impairment of the normal global, synchronized activity that follows processing of a salient visual stimulus and causes visual consciousness. Importantly, we believe this damage does not completely ‘shut down’ the communication between the central hubs of the GWS and the visual system, because (i) damage is not complete and/or (ii) plasticity mechanisms may come into play and partially restore function. Indeed, as defended by Hadid and Lepore (2017), the remaining connections enable some residual processing, and depending on which connections (short-ranging only or some more long-ranging) different remaining behaviours can be observed. From this perspective, we believe it is important to focus on the nature of visual awareness in HH, specifically on the fact that it is ‘different’ from normal visual awareness. We defend that it is thus likely to rely on different pathways than in neurotypicals. Furthermore, we believe it relies on a separate route from the one subserving objective residual capacities. More precisely, objective residual capacities would be mediated by subcortical routes to functional extrastriate areas, referred to as the feedforward sweep in RPT. The interpretation of visual stimulation in the blind VF through this route relies on the intrinsic capacities of neurons in these areas to respond to stimuli and be tuned to specific features. Different patients may exhibit different residual objective capacities depending on which routes are still functional after the lesion to V1. On the other hand, subjective residual capacities perhaps rely on remaining connections from the extrastriate areas and/or subcortical areas to the prefrontal areas, as argued by the GWS. As previously mentioned, prefrontal areas are implicated in metacognition and believed to be involved in awareness. If there are functional visual routes that can transmit information to prefrontal areas, it is thus likely to stimulate these functions. Seeing the disruption of awareness caused by a V1 lesion, V1 is necessary for normal awareness. Nonetheless, this does not mean it is the only actor in generating awareness. Perhaps it is necessary in itself and as it provides connections to prefrontal areas. We propose that without V1, prefrontal areas may still receive enough input from the visual system (via subcortical and/or extrastriate connections) to generate some sort of sensation, which can result in an accurate subjective report (when given the appropriate response scale). V1 is necessary for normal visual awareness, yet prefrontal activation can be sufficient for generating a sense of visual feeling. The behavioural observation of subjective sensitivity in hemianopics is inherently different from visual consciousness; it is, in fact, the manifestation of visual information reaching prefrontal areas and is translated to a report of a ‘sense of awareness’. This would explain why the phenomenological experience of subjective perception in the blind VF cannot be qualified as normal vision (even degraded normal vision).

By this account, blindsight results from partially preserved function within the visual system, with no connexion to the GWS (Fig. 3 left), while blindsense results from a remaining connection between altered yet functional visual areas and the attentional system (Fig. 3). Indeed, in blindsense, some subjective experience remains in response to visual stimulation, yet it is not interpreted as visual experience; this might result from the fact that the attentional system detects a presence and activates the GWS, but the representations that enter the GWS are only ‘attentional’ (‘there is something’) and cannot be corroborated by strong visual representations due to lack of recurrent connections to V1, hence the interpretation that ‘there is something but it is not visual’. The more long-ranging connections between these workspaces are preserved (architecturally and functionally, i.e. synapses, temporal synchrony, etc.), the more phenomena such as those that constitute blindsight and blindsense can be observed in laboratory settings. These however do not spontaneously generate visual awareness in the daily life of patients perhaps because of the extremely conservative perceptual criterion (Ko and Lau 2012; Morales *et al.* 2019), which seemingly results from becoming blind in half the VF. The remaining long-range connections are not sufficient to cause a strong enough global ignition of the consciousness workspace to override the expectation that no visual stimulation will happen in the blind hemifield.

**Figure 4.**
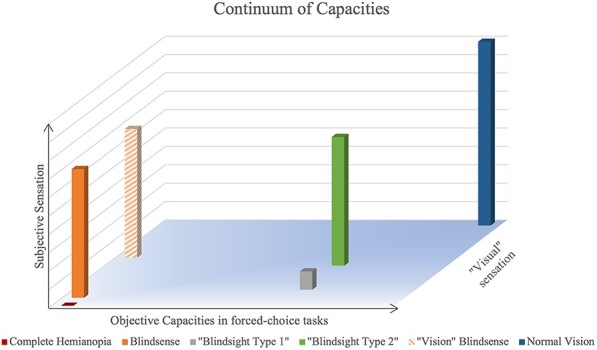
Illustration of the spectrum model in hemianopic patients. Capacities of the patients in their blind hemifield are represented through three axes: subjective sensation (i.e. feeling something), objective capacities (i.e. discriminating two form of a stimulus) and ‘visual’ sensation (i.e. seeing the stimulus). Six cases described in the literature are reported on this model: complete hemianopia, blindsense and ‘vision’ blindsense, type 1 blindsight, type 2 blindsight and ‘visual’ sensation

By conceptualizing ‘blindsight behaviours’ in consonance with theories of consciousness, we propose that the two opposing models (degraded vision versus unconscious vision) can be reconciled: the various behaviours described are evidence that some visual information processing is operational in hemianopics with varying levels of conscious access to this processing (Fig. 4). These different levels of access create a ‘spectrum’ of atypical behaviours across these patients, which could be organized along three dimensions, as described in Fig. 4. The spectrum of blindsight is determined by the extent of damage’s consequences, which varies between individuals, and, within each patient, by the many factors that can influence if a stimulus can elicit a conscious phenomenal experience at a given time (which would be varyingly different from normal vision due to the damage—see Fig. 4). As previously mentioned, a characteristic of blindsight behaviour is that performance on two-interval forced-choice visual discrimination tasks is better than on YN visual detection tasks (Azzopardi and Cowey 1997). However, one could argue that these tasks are neither similar nor comparable at all. We defend that in blindsight patients, it is fathomable that capacities for differentiating two stimuli (i.e. 2AFC) and for detecting a stimulus versus nothing (i.e. YN) could be affected differently, as these tasks are not in essence identical, even in neurologically normal subjects (Lee *et al.* 2018). A discrimination task is akin to recognition; it necessitates differentiation of stimulus features. This relies on higher-order processing and thus could generate stronger signalling from visual areas to prefrontal areas, amplifying the visual signal’s salience according to the GWS.

Crucially, we believe this level of conscious access can be modulated by various factors linked to the conditions of the visual experience, thus creating a spectrum from unconscious to conscious perception ‘within’ each patient. Interestingly, this conceptualization of how ‘blindsight’ behaviour results from post-chiasmatic structural damage altering GWS connections and dynamics also allows theorizing how the other functional changes seen in HH patients could arise. Hallucinations, anosognosia (lack of awareness of one’s own deficit) and sightblindness (subtle ipsilesional hemifield impairments) are alterations of perceptual experience also recurrently observed in HH patients (see Chokron *et al.* 2020 for review). Although beyond the scope of this paper, we believe that in the same way that remaining processing units and connections subserve residual processing in the blind VF, it is also the precise remaining network that enables these other dissociations to occur. We detail below both extrinsic and intrinsic factors at play in the phenomenological experience of one unique patient.

### Modulators of the perceptual spectrum in hemianopic patients

#### Extent of structural damage and its functional consequences

The most obvious influence of the damage on its behavioural consequences is necessarily the actual extent and localization of said damage. Indeed, it seems quite self-explanatory that the remaining functionality depends on the exact impaired and intact neuronal connections. However obvious this concept might be, it is quite difficult to have an idea of the extent of an infarct, especially of whether certain pathways remain (partially) functional. It is inherently difficult to investigate the effect of lesion characteristics on ‘blindsight’ occurrence at group levels; however, some studies have aimed to investigate the effect of lesion localization or size on the behavioural consequences of post-chiasmatic damage. Cavézian *et al.* (2015) considered whether the side of HH-causing lesion had an impact on processing in the sighted VF, finding that right brain damage appears to have more detrimental consequences. A study on post-stroke hallucinations uncovered an inverse correlation between lesion size and hallucination occurrence (Martinelli *et al.* 2020). The GWS highlights the importance of synchronous activity for phenomenological consciousness, thus implying HH patients without awareness in their blind VF have asynchronous activity. Indeed, many have suggested damage in hemianopics results in a global asynchrony along the visual pathway (Melloni *et al.* 2007). Moreover, by using TMS, Silvanto *et al.* (2007) created bilateral-MT synchrony in patient GY, successfully triggering the perception of phosphenes in his blind VF. This suggests that if damage impairs synchronous activity in the brain, it is likely to impair awareness as well.

Studies of the underlying neuroanatomy of blindsight have provided clues as to which remaining processing units subserve the different residual capacities. As previously mentioned, the pathways to extrastriate cortex bypassing V1 seem essential (Danckert and Rossetti 2005). For instance, Ajina *et al.* (2015) showed through tractography analysis that connections from LGN to extrastriate areas are essential for it to occur. In addition, the importance of SC activity has also been highlighted, notably as much of the post-stroke plasticity strengthens and regenerates its connectivity (Leh *et al.* 2006). Evidence that the extent and strength of connections influences where along the spectrum of residual processing one is located also comes from primate studies. Indeed, the fact that training monkeys with visual cortex ablation resulted in visual capacities suggestive of higher-order processing (Humphrey 1974) indicates that contralesional hemifield processing capacities are modulated by post-stroke plasticity, itself modulated by experience. In corroboration with this finding in primates, the intensive visual training—without feedback—of patients in their blind VF led to an increase in detection and/or subjective awareness of stimuli as well as some occurrences of improvement in the Humphrey perimetry test (Sahraie *et al.* 2006, 2013), suggesting that visual stimulation in the field defect can drive retinotopic remodelling to overcome the lesion to a certain extent.

Brain function cannot be solely explained through the state of its structural arrangement; we must also consider the level of activity and operativity of the neuronal networks.

#### Baseline activity and prior belief

If the baseline activity is heightened relative to normal baseline levels, the amount of activity necessary to reach the threshold for global activation is correspondingly reduced. The baseline activity of the networks and workspaces could be enhanced by specific instructions calling for these patients to expect feeling, sensations, awareness or even vision. Such an increase in baseline activity along the concerned networks could be the reason why certain visual stimulations in laboratory conditions reach the threshold for perception (Sergent and Dehaene 2004b). The degraded vision theory postulates that patients retain a form of vision, but they just do not report it because of a highly conservative criterion. Perhaps, this is correct, but this abnormally high criterion to report visual sensation is in itself interesting and has to be explained. First of all, the fact that reporting a perception requires insistent prompting of such a sensation, in lab conditions, suggests it must be different from conscious vision. Perhaps, this highly conservative ‘response’ criterion results from the fact that the damage has resulted in a vastly reduced probability that local loops of activity in the visual cortex areas are strong enough to evolve into more global loops of activity or that the global activity is somewhat abnormal. Because of that, even at times where, by chance, there is sufficient local activity to cause ignition of some more global loop, this is interpreted as an ‘error’, as it might correspond to spontaneous firing with no external cause. Conversely, damage to the visual pathway can result in an impairment of inhibitory control to extrastriate areas (Abrams *et al.* 2004), which is a proposed mechanism of how spontaneous firing may generate visual hallucinations in cortically blind patients (Martinelli *et al.* 2020).

Hemianopia’s defining symptom is cortical clinical blindness in the hemifield contralesional to the damage. This translates into hemianopics lacking vision in half of their previously normal VF. Patients often describe they conceptualize the blind VF as the back of their head, i.e. their VF is smaller than prior to the damage but this is not something they are constantly aware of. If we reason in the Bayesian framework, the prior belief relative to the visual stimulation that can be received in such a place is that there can be none. We can well imagine this prior belief to be quite strong. Thus, in a normal, everyday context, any activity relative to visual stimulation in the blind hemifields, even if it is truly the result of visual stimulation, will be overridden by this strong prior belief. However, laboratory conditions can modulate the prior belief in tested individuals: the mere fact that these patients are brought in for studies of their visual capacities in their blind VF incites them to believe they perhaps have some remnant abilities; the instructions often include researchers insisting the patients report ‘any’ sensation.

#### Extrinsic factors

The difficulties in measuring consciousness have been overcome by designing paradigms that assess components of consciousness in a very specific manner. As a result, the other components of consciousness, which are not the focus of a paradigm, can be understated or even muted during these assessments. In the framework of blindsight, this translates into the fact that the administration of different tasks will inherently lead to different performance of patients with a blind VF (Stoerig 1996; Danckert and Goodale 2000; Danckert and Rossetti 2005). For instance, forced-choice tasks with many trials exacerbate the capacity to perform discrimination based on perceptual characteristics of a stimulus whilst implicit paradigms can emphasize covert spatial orienting and motion detection. We believe the nature of stimuli (size, contrast, etc.), their duration as well as the wording of the task can all influence the performance of an HH patient observed in lab conditions. For instance, if a stimulus overlaps both the sighted VF and the blind VF, it is likely that this will facilitate visual tasks relating to this stimulus (e.g. see Jackson 1999). Conversely, if the stimulus’ protrusion is incoherent/aberrant with the part in the sighted VF, we postulate this will create a greater proportion of mistakes relating to the stimulus. Furthermore, tasks can rely on automatic behaviour, or higher levels of processing and depending on which residual pathways remain in a patient, the type of task could lead to different ‘performance’ in one same individual. It is thus important to nuance the attribution of ‘blindsight’ designation, which is why we believe the notion of a spectrum is more appropriate than the notion of categories of patients.

In order to study the subtly different manifestations of processing in the blind VF of patients, sensitive and precise measures are essential. Assessing the level of conscious access to a visual percept has the inherent difficulty that we can only rely on reports. It is best capitulated by forced-choice discrimination paradigms in the framework of SDT, which quantifies sensitivity *d*ʹ as well as response bias (or decision criterion). SDT mathematically dissociates sensitivity from decision criterion, which is crucial in these patients who tend to have high criteria (i.e. are less likely to report visual perception when they are not sure; Ko and Lau 2012). To best dissociate objective and subjective performance, the task should be split into (i) a part without mentions of perception (e.g. ‘Was the patch’s orientation to the left?’ or ‘Was there an “X”?’) and (ii) a part emphasising subjective phenomenology during the trial by explicitly mentioning sensation/awareness (e.g. ‘Did you feel the patch was oriented to the right?’ or ‘Were you aware of an “O”?’). In both, response can be prompted with a binary or graded scale. The measure of subjective awareness is more sensitive when being assessed by a graded scale, as is shown by differential diagnosis of patient GR as blindsight type I or II depends on whether awareness was assessed with a binary or graded response (i.e. PAS; Ramsøy and Overgaard 2004). Furthermore, as revising the PAS to create the SAS led to the discovery of blindsense (Garric *et al.* 2019), we believe it is more adapted to the phenomenological experience of these patients and should therefore be preferred when assessing HH patients. The PAS is designed to measure perceptual consciousness in the modality of the sense of interest (here vision) in a healthy subject, irrespective of whether this subjective perception is used for task performance (Dienes and Seth 2010). The SAS is the adaptation of this scale to individuals with brain damage causing a loss in the modality of interest; it is designed to be more sensitive to the less salient levels of perceptual awareness, which are of utmost interest in cases where a perceptual modality is thought to be lost. Such sensitivity is necessary: Mazzi *et al.* (2016) measured their subject’s capacities as being ‘at chance’ in discrimination despite the subject in fact ‘reporting’ some ‘brief glimpses’ during stimulus trials when given a scalar response option. The necessity in such a sensitivity is obvious when considering that Mazzi *et al.* (2016) report their subject as being ‘at chance’ in discrimination, despite the subject having reported ‘brief glimpses’ in trials which stimuli. This was potentially an overlooked case of blindsense. We believe these weak subjective perceptions are indications of a potential improvement of the visual abilities of these patients, and the important identification of blindsense has highlighted that they may occur despite no objective sensitivity. Whichever methodology is used to assess visual processing in the blind VF, it is essential that the interpretation of the measures remains specific to what is being assessed and that researchers are cautious to avoid over-generalization. One way to overcome this would be to administer complementary tasks, perhaps in different sessions, to the same participants.

### Predictions of the theory

Several predictions arise from conceiving residual processing in the blind VF as a spectrum of deficits across the three dimensions of objective discrimination abilities, subjective experience and subjective ‘visual’ experience (Fig. 4). First and foremost, we believe that if thorough perceptual protocols are administered consistently to HH patients, far more profiles will be uncovered. Each patient will subtly vary in the subjective access to perception, in the objective perceptual capacities and in the visual degree of the perceptual experience (Fig. 4). To test whether there is indeed a spectrum ‘within’ each patient, administering visual tasks of different designs should lead to different ‘performance’, as task design can modulate expectations and baseline activity of specific networks. The differential diagnosis of patient GR as blindsight type I or II depending on whether awareness was assessed with a binary or graded response (i.e. PAS; Ramsøy and Overgaard 2004) supports this idea. It remains crucial to consider criteria measurements throughout these different tasks, as it is conceivable shifts occur between the tasks and modulate the response pattern.

As mentioned, we believe prior belief modulates the potential salience of stimuli in the blind VF. We therefore predict that pre-briefing one of two groups of naïve HH patients by informing them of implicit perceptual abilities in the blind VF will lead that group to perform better (note that by naïve we mean unaware of the existence of blindsight or related phenomena).

Metacognition is one’s assessment of the correctness of one’s perceptual decisions. Metacognition is believed to rely on anterior prefrontal cortex (McCurdy *et al.* 2013), thanks to its input from the dorsal and ventral visual streams (Young 1992), making long-range connections crucial. If residual processing is a spectrum from basic objective perceptual capacities to phenomenological experience of stimuli in the blind VF associated with an increase in inter-hemispheric connections (Leh *et al.* 2006) and prefrontal activations (Sahraie *et al.* 1997), metacognitive capacities should be better in patients sitting on the latter end of the spectrum. We therefore hypothesize metacognitive efficiency will be more preserved in patients showing greater awareness of stimulations in their blind VF.

Another theoretical outcome of this theory is that hemianopic patients should be able to move along the perceptual spectrum, providing there is strengthening of the remaining connexions. In this way, the spectrum capitulates the temporal dynamics of the evolution of blindsight behaviour during stroke recovery. For instance, training the blind VF (Chokron *et al.* 2008; Trevethan *et al.* 2012) could potentiate plasticity mechanisms, which in turn would strengthen the long-ranging connections necessary to trigger increasing levels of awareness. Indeed, stimulation of the blind hemifield in patients with ‘blindsight’ appears to favour (some) restoration (Chokron *et al.* 2008). In the same vein, we predict little or no blind-VF processing capacities will be apparent when assessing acute post-stroke patients (about 2 weeks; Coleman *et al.* 2017), as cortical reorganization would not yet have occurred.

Regarding cortical reorganization and visual recuperation in patients with VF defect, several authors have used animal models, in particular in marmosets (Hagan *et al.* 2017), to investigate the effect of age at the lesion acquisition as well as delay from it on the temporal dynamics of visual recuperation. First, Rosa *et al.* (2000) studied cell responsiveness shortly after V1 lesion in marmosets, showing MT neurones with receptive fields in the scotoma were responsive and direction selective. On the other hand, Collins *et al.* (2003) found no significant responsiveness in a similar study. The main difference between the two studies is that Collins et *al.* studied monkeys ‘straight after’ the lesion, which is coherent with previous prediction. Conversely, Hagan *et al.* (2020) recorded the response properties of MT neurons to random dot stimuli in marmoset monkeys not immediately after the lesion but 7–11 months following it. These authors confirm the prediction that MT neurons still respond to random dot patterns long after V1 lesions, although they demonstrated that the prevalence of direction selectivity was significantly reduced and the sensitivity to motion coherence was significantly altered. In addition, these authors were able to demonstrate that the observed changes in neural responses were consistent with underlying changes in inhibition and lateral connectivity. As these authors underline, in marmoset, MT neurons seem to continue to respond shortly after V1 lesions, but in humans, clinical work has shown that lesion effects can take up to 6 months to stabilize. However, the plasticity mechanisms might already be triggered during the acute post-stroke stage (Su and Xu 2020), hence we believe the potential for improvement of sensitivity in the blind VF may be greatest if stimulation is initiated then (Saionz *et al.* 2021).

Using a complementary approach, Sanchez-Lopez *et al.* (2020) have studied the neuroanatomical bases of the unconscious above-chance performance and of the phenomenological aspects that may be associated. These authors tested 17 hemianopic patients with movement and orientation discrimination tasks over visual gratings presented to the sighted or blind hemifield. Patients were then classified into four groups on the basis of the presence or absence of above-chance discrimination, with or without reported perceptual awareness for the stimuli presented to the blind hemifield. In the four groups, the authors carried out analyses of lesion extent of various cortical areas, probabilistic tractography as well as assessment of the cortical thickness of the intact hemisphere. The two areas that turned out to be critical for above-chance performance in discriminating moving versus non-moving visual stimuli were the precuneus and the posterior cingulate gyrus while for perceptual awareness reports the crucial areas were intracalcarine, supracalcarine, cuneus and the posterior cingulate gyrus. Interestingly, this approach allowed demonstrating that the proportion of perceptual awareness reports was higher in patients with a spared right hemisphere. As to probabilistic tractography, all pathways examined yielded higher positive values for patients with perceptual awareness reports. Finally, the cortical thickness of the intact hemisphere was greater in patients showing above-chance performance than in those at chance. Along those lines, it would be highly interesting to use a similar approach as Sanchez-Lopez and colleagues (2020) and study the neuro-anatomical correlates of different patterns of responses while taking into account both objective and subjective perception in the blind VF using the SAS, as well as factoring in the delay from the lesion acquisition.

Although evidently not an exhaustive list, we believe testing the predictions mentioned here would offer valuable insight into the underlying mechanisms of residual processing in the blind VF of hemianopic patients.

## Conclusion

We provide a unifying explanation of the theoretical conceptualization of blindsight behaviour mechanisms. It has proved difficult to qualify the experience of blindsight as degraded normal vision, as there is overwhelming evidence of qualitative differences between blindsight experience and normal vision (Weiskrantz 2008). We defend that a wide multidimensional spectrum of deficits from total loss of sensory information processing in the visual modality to abnormal visual sensation best describes the different phenomenological profiles observed in HH patients. It appears that a non-negligible proportion of individuals with a blind VF show some evidence of residual processing in their visual modality, differences lying in the extent to which they can achieve explicit access to these remaining visual representations. The said differences can be found not only between individuals but also within an individual, depending on various parameters, as described in the previous section. Moreover, ‘blindsight behaviour’ can shed light on how normal visual consciousness arises. Identifying the functioning versus the damaged neural components of the visual system in blindsight patients will provide clues relative to the neuroanatomical correlates of consciousness. We believe these behaviours can find plausible mechanistic counterparts within the framework the GWS theory of consciousness.

Understanding the ‘blindsight’ behaviours might serve three important goals. Firstly, understanding a pathology can result in a better therapeutical approach. For instance, visual stimulation in the blind VF of HH patients has already shown evidence of therapeutical potential, and a more precise stimulation of the blind VF is likely to be developed with a better understanding of the residual phenomena. Second, the various dissociations observed between objective performance and phenomenological experience in these patients invite us to reconsider how visual consciousness arises in the brain. In the same vein, knowing which processes and connections support blindsight behaviour undeniably would provide valuable information as to the neuroanatomical basis of consciousness, both pathological and normal consciousness. Last but not least, the evolution of blindsight behaviours within each patient will give insight into the plasticity of consciousness and how it may be malleable to a certain extent.

Further research remains crucial to further elucidate the precise underlying mechanisms of blindsight behaviours, with thorough and reliable methodology. More group studies are essential in order to estimate properly the frequency of these types of behaviours. We suggest these studies should range over all levels of cognitive levels, from first-order perception to metacognition, in order to get the full scope of the residual activity along the neural components of conscious experience, after the damage of the primary visual pathway.
